# Changes in childhood vaccination coverage over time in the Democratic Republic of the Congo

**DOI:** 10.1371/journal.pone.0217426

**Published:** 2019-05-24

**Authors:** Vivian H. Alfonso, Anna Bratcher, Hayley Ashbaugh, Reena Doshi, Adva Gadoth, Nicole Hoff, Patrick Mukadi, Angie Ghanem, Alvan Cheng, Sue Gerber, Guillaume Ngoie Mwamba, Jean Jacques Muyembe Tamfum, Emile Okitolonda Wemakoy, Anne W. Rimoin

**Affiliations:** 1 Department of Epidemiology, University of California Los Angeles, Los Angeles, California, United States of America; 2 McKing Consulting, Atlanta, Georgia, United States of America; 3 National Institute for Biomedical Research (INRB), Kinshasa, Democratic Republic of the Congo; 4 Bill and Melinda Gates Foundation, Seattle, Washington, United States of America; 5 Expanded Programme on Immunization, Kinshasa, Democratic Republic of the Congo; 6 Kinshasa School of Public Health, Kinshasa, Democratic Republic of the Congo; University of Campania, ITALY

## Abstract

Despite increased vaccination rates, the burden, morbidity and mortality associated with vaccine preventable diseases remains high. In the Democratic Republic of the Congo (DRC), potentially unreliable data and geographically varied program provision call for a better understanding of vaccination coverage and its changes over time at the country and province level. To assess changes in the proportion of children who were fully vaccinated over time in the DRC, vaccination histories for children 12–59 months of age were obtained from both the 2007 and 2013–2014 Demographic and Health Surveys (DHS). Changes were assessed, both at the country- and province-levels, to identify potential geographic variations. Vaccination coverage improved 70% between the DHS waves: 26% compared to 44% of 12–59 month-old children met full vaccination criteria in 2007 and 2013–2014, respectively (n_2007_ = 3032 and n_2013-14_ = 6619). Similarly, there was an overall trend across both DHS waves where as year of birth increased, so did vaccination coverage. There was geographic variation in immunization changes with most central and eastern provinces increasing in coverage and most northern, western and southern provinces having decreased vaccination coverage at the second time point. Using nationally representative data, we identified significant changes over time in vaccination coverage which may help to inform future policy, interventions and research to improve vaccination rates among children in the DRC. This study is the first of its kind for the population of DRC and provides an important initial step towards better understanding trends in vaccination coverage over time.

## Introduction

Vaccinations are one of the most efficient and cost-effective public health interventions, resulting in significant reductions in global child morbidity and mortality over the past five decades [1,2]. Child deaths, due largely to infectious diseases, dropped from 12.7 million in 1990 to 5.9 million in 2015 [3], and it is estimated that two to three million child deaths are averted each year due to vaccination [4]. Despite such improvements in global immunization coverage, the burden of vaccine preventable diseases (VPDs) remains high in low- and middle-income countries: VPDs are responsible for 1.5 million preventable deaths in children under the age of five [4] and approximately 29% of deaths among children one to 59 months of age [5]. The 2005 Global Immunization Vision and Strategy set the goal for all countries to meet and sustain 90% national of diphtheria-tetanus-pertussis (DTP3) coverage by 2015 [6]. However, by 2017, 19.9 million children were not fully vaccinated with DTP3 and the majority lived in 10 countries: the Democratic Republic of the Congo, Afghanistan, Angola, Ethiopia, India, Indonesia, Iraq, Nigeria, Pakistan and South Africa [4].

In the World Health Organization (WHO) African region, child immunization has increased despite financial, geographic and administrative obstacles [7]. Coverage of one dose of measles vaccine administered through routine immunization program, as estimated by WHO/United Nations International Children’s Emergency Fund (UNICEF), increased from 53% in 2000 to 70% in 2017 [8–10]. However, VPDs remain widespread [11], which may be due to difficulties in organization and coordination of vaccine supplies, inadequate maintenance of cold chain, vaccine hesitancy, and reliance on external international funding [8,12,13].

In the Democratic Republic of the Congo (DRC), there are an estimated 11.9 million children under the age of five and implementation of an effective Expanded Program on Immunization (EPI) has been challenging [8]. The Congolese EPI is responsible for creating and executing an Annual Operating Plans and Comprehensive Multi-Year Plans, generally covering five years, that address all immunization activities in the DRC. For example, the DRC’s EPI strategic plan calls for a single dose of measles containing vaccine at 9 to 11 months of age, and a second dose given through campaigns targeting children that are 6–59 months of age.[14] In addition to generating these plans, the Congolese EPI oversees immunization campaigns, strengthens routine immunization, introduces new vaccines, delivers community mobilization campaigns for vaccination, and improves VPD surveillance.[15]While one objective of the Congolese EPI is vaccination of 93% of children with 3 doses of DTP vaccine before their first birthday [16], lack of infrastructure and difficult terrain isolate many villages in DRC, making vaccine supply, delivery, and proper storage difficult [8].

Despite such challenges, according to official country estimates coverage with a third dose of DTP vaccine in DRC increased from 40% in 2000 to 94% in 2017 [17,18]. However, the accuracy of these estimates are called into question as the Service Readiness and Availability assessment in 2014 found that while 75% of all facilities offered vaccination, only 42% of these facilities had the necessary equipment or personnel available on the day of the assessment [19]. Furthermore, armed conflict has impacted the country for decades, and reports suggest that both short-term [20] and prolonged interruptions in vaccination activities due to violence and unrest may result in increased child mortality and significant immunity gaps in both older children and adults [21,22]. Furthermore, official country estimates identified that coverage with a routine first dose of measles vaccine increased from 46% in 2000 to 92% in 2017; however, DRC has experienced large-scale outbreaks of measles in recent years [14,23]. Although DRC has successfully interrupted wild poliovirus transmission, the risk of an outbreak in DRC remains high [24]: multiple, independent emergences of vaccine-derived type 2 poliovirus cases have been confirmed in the country as recently as October 2018 [25,26]. This persistence of VPDs calls into question the accuracy of vaccination estimates based on administrative reports, which have been consistently higher than WHO/UNICEF estimates since 2000 [17].

Given the importance of accurate estimates of vaccination coverage in the prevention of disease, an assessment of vaccination status in the DRC is necessary to estimate population immunity changes over time. Knowledge of these changes could identify previous successes and areas in need of additional vaccine delivery programs in the DRC [8]. Therefore, we characterized vaccination status using nationally representative data from the 2007 and 2013–2014 DRC Demographic and Health Surveys (DHS). We assessed vaccination status for children from 12 to 59 months of age and identified changes over time. While efforts to improve vaccination coverage to meet regional and global targets are underway, this information will be useful in understanding trends of vaccination in the DRC and may inform future policy and financial decisions.

## Methods

### Study population

The sampling design and data collection procedures of the Demographic and Health Surveys (DHS) have been described elsewhere [27–30]. Briefly, they are cross-sectional, household-based, surveys used to generate nationally representative data for monitoring population health and social indices; surveys are typically conducted on a rolling cycle approximately every five years. All households in DRC were eligible for these surveys and were chosen by a two-stage stratified cluster sampling design. We utilized data from both the first and second waves of the DRC DHS, administered from January to August 2007 and November 2013 to February 2014, respectively.

Data were collected from 8,886 (out of 8,945 eligible and 18,171 (out of 18,190 eligible) households in 2007 and 2013–2014, respectively. According to the design of the DHS, all children 0 to 59 months of age living in households in which the biological mother was present and consented were eligible to participate (n = 3,951 from the first wave and n = 8,552 from the second wave). Information collected on participating children included, but was not limited to, socio-demographic information, anthropometric measures, and vaccination history. All survey data was double entered from paper questionnaires to an electronic format by the DHS Program, using the Census and Survey Processing System (U.S. Census Bureau, ICF Macro), and verified by comparison.

### Outcome assessment

Child vaccination status was assessed according to the following vaccination schedule for the DRC: Bacille Calmette-Guerin (BCG) vaccine at birth; oral polio vaccine at 0, 6, 10 and 14 weeks (OPV 0–3); diphtheria, tetanus, pertussis vaccine at 6, 10 and 14 weeks (DTP 1–3); measles vaccine at 9 months; and yellow fever vaccine at 9 months (Table 1) [31]. Children greater than 12 months of age reporting BCG, OPV1-3, DTP1-3, measles and yellow fever at the time of the survey were categorized as fully vaccinated, regardless of age at the time of administration. Pneumococcal conjugate (PC 1–3), pentavalent diphtheria, tetanus, pertussis, *Haemophilus influenza* type b, hepatitis B vaccine (DTPwHibHepB 1–3) and Inactivated Polio Vaccine (IPV) vaccination status were not included in this definition as they were introduced after the 2007 DHS,. Children with any combination other than the full course were categorized as partially immunized and children reporting no vaccinations were indicated as non-immunized.

**Table 1:** Recommended vaccine administration schedule for children in the DRC according to the World Health Organization (WHO) [31], 2017.

		Birth	6 weeks	10 weeks	14 weeks	9 months
Bacille Calmette-Guerin vaccine		**x**	** **	** **	** **	** **
Oral polio vaccine	OPV 0	**x**	** **	** **	** **	** **
	OPV 1	** **	**x**	** **	** **	** **
	OPV 2	** **	** **	**x**	** **	** **
	OPV 3	** **	** **	** **	**x**	** **
Inactivated polio vaccine IPV		** **	** **	** **	**x**	** **
Diphtheria, tetanus^2^, pertussis, *Haemophilus influenza* type b, and hepatitis B vaccine	DTwPHibHepB 1	** **	**x**	** **	** **	** **
	DTwPHibHepB 2	** **	** **	**x**	** **	** **
	DTwPHibHepB 3	** **	** **	** **	**x**	** **
Pneumococcal conjugate vaccine	PC 1	** **	**x**	** **	** **	** **
	PC 2	** **	** **	**x**	** **	** **
	PC 3	** **	** **	** **	**x**	** **
Measles vaccine		** **	** **	** **	** **	**x**
Yellow fever vaccine		** **	** **	** **	** **	**x**

### Statistical analyses

Analyses were limited to children 12 to 59 months of age as children should be fully vaccinated by this age according to the WHO recommended schedule. To assess changes in vaccination coverage across the entire nation, we assessed differences in vaccination status for each vaccine and full, partial, or no immunization across the two waves of the survey using Rao-Scott chi-square analyses. Furthermore, as mothers of the youngest children are most likely to accurately recall vaccination history and the youngest children were most likely to have a vaccination card at interview compared to other age groups, we additionally assessed vaccination coverage among children 12 to 23 months of age in order to control for recall bias.

To explore the relationship between age and vaccination status, percent of fully vaccinated children was plotted against birth year to assess for possible cohort effects of vaccination status. The results for this relationship are displayed for two populations: first, for children with a vaccination card whose vaccination status was determined from that card, and then for all children with vaccination status coming from any source of information (maternal recall and vaccination card). Significance was tested with a Mantel-Haenszel Chi-Square test for trend.

Additionally, to assess spatial distribution and changes in vaccination status over time among 12–59 month old participants, maps of full vaccination status were created for the first (2007) and second (2013–2014) DHS, separately, as well as the change over time within each province. Change over time was calculated as follows:

$$\frac{\left((\%\;fully\;vaccinated\;in\;2013-2014)-(\%\;fully\;vaccinated\;in\;2007)\right)}{\left(\%\;fully\;vaccinated\;in\;2007\right)}\times 100$$

All analyses were performed using SAS software, Version 9.4 (SAS Institute, Cary, NC) and maps were generated using ArcGIS software version 10.5 (ESRI, Redlands, CA).

### Ethical approval

Ethical approval was obtained at UCLA Fielding School of Public Health and the Kinshasa School of Public Health. Informed consent was obtained from all enrolled participants as a part of the DHS survey.

## Results

Among children 12–59 months of age, significant increases in BCG, OPV1-3, DTP1-3, measles, and yellow fever vaccinations were observed between the 2007 and 2013–2014 surveys (Table 2). As a result, the proportion of children who were fully vaccinated significantly increased between the two waves from 26% in 2007 to 44% in 2013–2014 (*p* < .0001). The percentage of children with no vaccinations decreased by 59% between the two surveys (17% in 2007 to 7% in 2013–2014, *p* < .0001). Additionally, vaccination card ownership changed over time; while the same proportion of biological mothers presented their children’s vaccination card during the two survey waves, reported ownership of vaccination card decreased from 2007 to 2013–2014 (61% and 51%, respectively, *p* = .001). Similar estimates of vaccination coverage was observed when the sample was restricted to 12–23 month-olds (S1 Table).

**Table 2:** Weighted vaccination coverage estimates for the democratic republic of the Congo demographic and health survey respondents 12–59 months of age by survey year.

	2007 (n = 3,032)		2013–2014 (n = 6,619)		
	n	% (95% CI)	n	% (95% CI)	*p-value*
Vaccination card^a^					
Yes, seen (1)	523	17 (14–21)	1,120	17 (15–20)	*0*.*0010*
Yes, not seen (2)	1,318	44 (40–48)	2,434	34 (34–41)	
No longer has card (3)	232	8 (6–9)	938	15 (13–17)	
No card (0)	940	31 (26–36)	1,950	30 (27–34)	
Reported vaccinations from vaccination card or maternal recall					
BCG^b^	2,224	74 (70–79)	5,417	84 (82–86)	*<0*.*0001*
Polio0^c^	1,320	44 (39–49)	3,006	47 (43–50)	*0*.*3484*
Polio1^d^	2,416	80 (76–84)	5,800	91 (90–93)	*<0*.*0001*
Polio2^e^	2,124	71 (66–75)	5,255	86 (84–88)	*<0*.*0001*
Polio3^e^	1,422	47 (43–52)	4,021	66 (63–69)	*<0*.*0001*
Polio vaccination completion (OPV1-3)^e^					
Full (1)	1,413	47 (43–51)	4,016	66 (63–69)	*<0*.*0001*
Partial (2)	996	33 (30–36)	1,576	26 (23–28)	
None (0)	595	20 (16–24)	518	8 (7–10)	
DTP1^f^	2,150	73 (68–78)	5,183	80 (78–83)	*<0*.*0001*
DTP2^g^	1,823	65 (57–67)	4,769	74 (71–77)	*<0*.*0001*
DTP3^g^	1,457	50 (44–55)	3,960	61 (58–65)	*0*.*0001*
DTP vaccination completion (DTP1-3)^g^					
Full (1)	1,449	49 (44–54)	3,955	61 (58–65)	*0*.*0001*
Partial (2)	702	24 (20–28)	1,230	19 (17–21)	
No (0)	788	27 (22–32)	1,254	20 (17–22)	
Measles^h^	2,072	70 (65–74)	4,918	76 (74–78)	*0*.*0062*
Yellow fever^i^	1,579	53 (48–58)	4,508	70 (67–73)	*<0*.*0001*
Vaccination status^j^					
Full (1)	753	26 (22–30)	2,644	44 (41–47)	*<0*.*0001*
Partial (2)	1,632	57 (52–61)	3,000	50 (47–53)	
None (0)	504	17 (14–21)	392	7 (5–8)	

^a^ 2007 n = 3013; 2013 n = 6441.

^b^ 2007 n = 2994; 2013 n = 6462.

^c^ 2007 n = 3016; 2013 n = 6464.

^d^ 2007 n = 3014; 2013 n = 6325.

^e^ 2007 n = 3005; 2013 n = 6110.

^f^ 2007 n = 2941; 2013 n = 6443.

^g^ 2007 n = 2938; 2013 n = 6439.

^h^ 2007 n = 2980; 2013 n = 6458.

^i^ 2007 n = 2985; 2013 n = 6436.

^j^ 2007 n = 2889; 2013 n = 6036.

**Fig 1:**
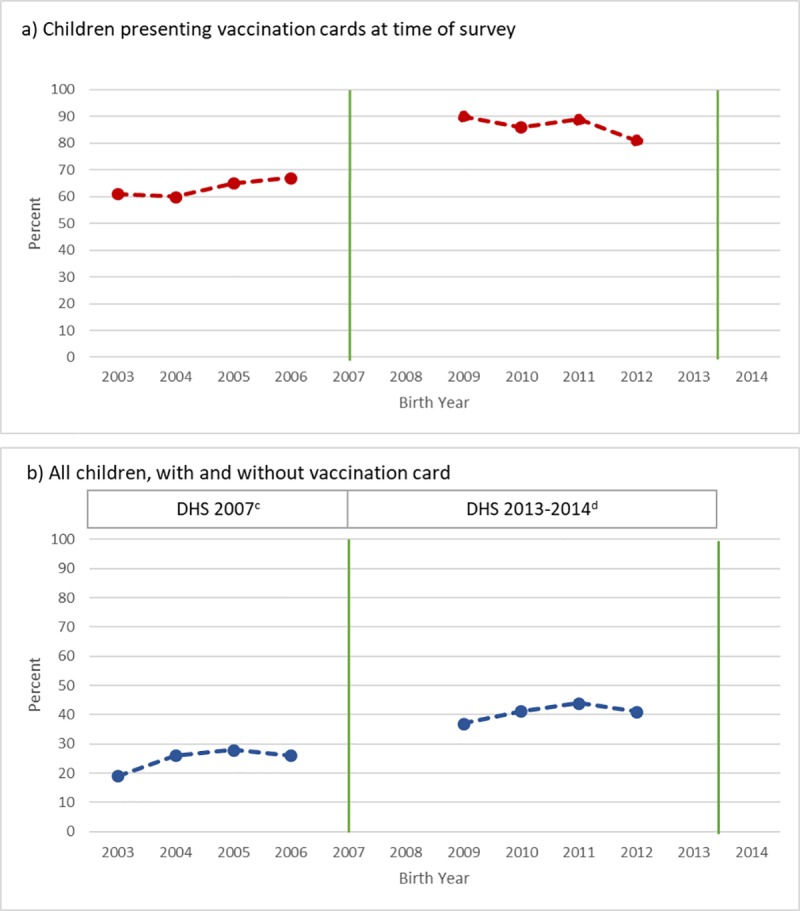
Percent fully vaccinated by birth cohort and source of information for 12–59 month old respondents of the 2007 and 2013–2014 DRC DHS. Blue lines represent the percent of children fully vaccinated as determined by all sources of information (maternal recall and vaccination card). Red lines represent full vaccination status among those with a vaccination card according to vaccination card only. Green lines represent the administration year of the two DRC-DHS survey waves. ^a^ n = 523; *p* for trend = .2442 ^b^ n = 1,120; *p* for trend = .0050 ^c^ n = 3,032; *p* for trend = .0033 ^d^ n = 6,619; *p* for trend = .0071.

To assess possible cohort effects, vaccination rate was plotted by birth year according to all sources of information and then restricted to children presenting vaccination card records at the time of interview (Fig 1). For all children in wave 1, younger children were more likely to be fully vaccinated compared to older children (26% born in 2006 vs 19% born in 2003, *p* for trend = .0033). A similar significant trend was observed for children in wave 2 according to birth year and full vaccination status (41% of those born in 2012 vs 37% of those born in 2009, *p* for trend = .0071). Among those presenting a vaccination card at the time of the second survey, 81% of those born in 2012 were fully vaccinated compared to 90% of those born in 2009 (*p* for trend = .0050). No findings were observed for wave 1 among those presenting a vaccination card during the interview (*p* for trend = .2442).

**Fig 2:**
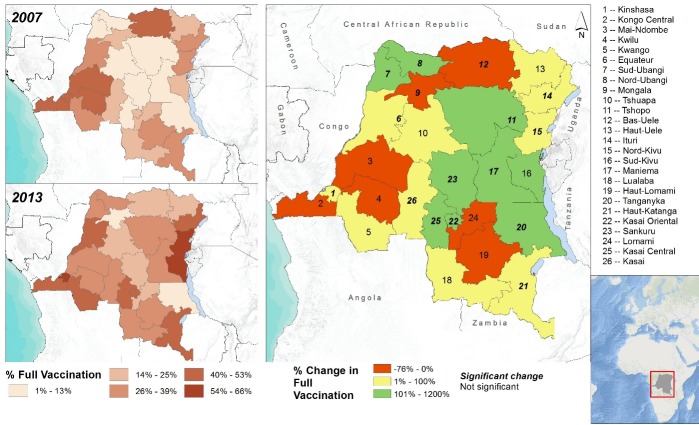
Spatial distribution of full vaccination status according to year (2007 and 2013–2014) and change in vaccination status over time among 12–59 month old DRC-DHS respondents. In the 2007 and 2013 panels, the darker the shade, the higher the proportion of fully vaccinated children. In the third panel, green represents a large increase in vaccination coverage (101% to 1200%), yellow represents moderate increase in vaccination coverage (1% to 100%) and red represents a decrease in full vaccination coverage (-76% to 0%) over time.

To assess geographic trends across survey waves, vaccination status (regardless of the information source) was mapped for the 26 provinces (Fig 2). In 2007, the provinces with highest percentage of fully vaccinated children included Mai-Ndombe (48%), Kongo Central (45%), Bas-Uele and Kinshasa (44%). In 2013–2014 survey, the provinces with the highest prevalence of full vaccination included Nord-Kivu (66%), Sud-Kivu (63%), Kinshasa (58%) and Kasai-Oriental (52%). While most provinces experienced an increase in vaccination status over time, a significant decrease in reports of full vaccination was observed for Mongala (-76%, *p* = .0099) and Bas-Uele (-68%, *p* = .0003) provinces.

## Discussion

In this analysis of health survey data from DHS 2007 and 2013–2014, we assessed changes in vaccination status among young children between 12 and 59 months of age in the DRC. Overall, improvements in vaccination coverage were observed over time: 26% compared to 44% of 12 to 59 month-olds were fully vaccinated in 2007 and 2013–2014, respectively. The biggest gains in coverage of individual vaccines were seen in OPV1-3, from 47% in 2007 to 66% in 2013–2014, and yellow fever, from 53% in 2007 to 70% in 2013–2014. This coverage is higher than some other developing nations such as Nigeria, where 23% of children were fully vaccinated in 2016 [32], but comparable to others such as India, where 43% of children were fully vaccinated in 2016 [33].

However, source of vaccination information and method of assessment is important for the interpretation of results because large discrepancies in vaccination estimates exist. In 2014, DTP3 coverage in DRC was estimated as 61%, 80% and 93% according to DHS, WHO/UNICEF and country official administrative estimates, respectively [17]. Only 17% of all children in this analysis presented their vaccination card at interview and reliance on maternal recall increased with child age, with a decrease in those presenting cards in the 2013–2014 DHS. Though information obtained from older children may be less accurate due to the lack of documentation and maternal recall, limiting analyses to the youngest age group (12–23 month-olds) produced the same findings as for the whole sample.

While we did not observe a difference in vaccination coverage reported through differing lengths of maternal recall, we did observe a large difference in vaccination coverage estimated through maternal recall versus vaccination card (Fig 1). However, it is difficult to draw conclusions from this finding as there are likely uncontrolled confounders that affect both vaccination status and vaccination card possession. For instance, a study examining measles vaccination utilizing the 2013–2014 DRC DHS data found that vaccination predictors and disparities, as well as data quality, varied by urban versus rural residence. The poorest rural children and most severely malnourished urban children were less likely to have been vaccinated for measles, and rural children less frequently possessed a dated vaccination card compared to urban children [34]. Previous research has also compared maternal reported vaccination status, vaccination card determined vaccination status, and serologically determined immunity for polio in the 2013–2014 DRC DHS data [35]. This research found a discrepancy between reporting sources and lab-confirmed immunity, where those reported as fully vaccinated according to vaccination card were more likely to be seropositive compared to those reported as such through maternal recall.

Our data showed trends in vaccination coverage by birth year (Fig 1). For all children in both the 2007 and 2013–14 DHS, vaccination coverage estimates were highest for younger children as reported by card and maternal recall; however, according to vaccination card for wave 2, older children were more likely to be vaccinated compared to younger children. Many factors may be influencing these observations. One possible reason is that there may have been decreased vaccination coverage over the span of birth years for children of 12–59 months of age in the 2013–14 DHS wave. This would lead to the observed pattern, by causing children who were born more recently to be less likely to have received a vaccine. Likewise, this pattern could also have been caused by an increased likelihood of experiencing catch-up vaccination with age. These catch-up vaccinations may be a result of routine mass immunization campaigns that are commonly held in DRC.[35] More research is needed to better understand these trends in vaccination by birth year.

Evaluation of spatial trends revealed that vaccination coverage varies significantly by geographic location. Our spatial analyses identified the northern, western and southern provinces as areas for future targeted efforts of more efficient and effective interventions to improve vaccination levels of young children in the DRC. In general, central and eastern provinces experienced improvements in coverage while northern, western and southern provinces were static or experienced declines in vaccination among children. Several factors may underlie geographic disparities in vaccination status, such as population density and topography. For example, increasing distance to health clinics, low health worker density, low availability of health facilities, type or quality of care received, affordability of vaccinations, and difficulty in accessing remote locations during vaccination campaigns by health workers may impact vaccination status of children [36,37]. These factors may affect change in vaccination over time either directly, through the variation in challenges faced over time, or indirectly, as resources to combat these challenges may be in flux.

Against expectations, yet in agreement with previous findings of measles vaccination in the DRC [34], our spatial analysis identified some eastern provinces that have consistently experienced armed conflict in the past two decades as areas that experienced increases in vaccination coverage. While public health improvements in conflict zones are not unheard of [38], this trend is in contradiction to much research that suggests armed conflict may inhibit vaccination activities [39–44]. Many factors, such as increased humanitarian aid, reduced severity of conflict, and mobility of individuals out of conflict areas into in more easily accessible areas for vaccination efforts, could have lead to higher vaccination coverage in these provinces. Given that our analysis does not speak to the etiologies of observed trends, more research is needed to identify the source of increased vaccination despite the presence of armed conflict in Eastern DRC.

This study is subject to several inherent limitations. Serologic confirmation of antigen exposure, vaccination, or immunity was not available for this analysis. However, immune reactivity has been studied for the 2013–2014 DRC DHS data for polio, measles and tetanus and results are presented elsewhere [34,35]. Only children whose biological mother was available to participate were captured by the survey; therefore, we were unable to assess trends in vaccination status for orphans, children living with other family members, or for children whose mothers did not participate. Additionally, survival bias may have played a role in our analysis: partially vaccinated or unvaccinated children may be more likely to die prematurely than fully vaccinated children; therefore, fully vaccinated children may have been overrepresented in our sample. While we identified province-level disparities in vaccination status of DRC children, finer geographic variations could not be assessed due to survey limitations. Finally, this is an analysis of two serial cross-sectional studies, and therefore we can only assess changes in prevalence and not incidence.

Despite its limitations, the DHS dataset is a repeated, standardized and highly detailed survey administered by an external organization, which allows for the assessment of vaccination data quality and coverage changes across all provinces of DRC [45]. Utilizing this data allows for a comprehensive review of the differences in vaccination rates over time. Furthermore, sensitivity analyses allowed for investigation of covariates impacting full vaccination status among the youngest children, which removed some of the bias (from recall or misclassification) that may have been present in analyses of the full sample.

Vaccines remain one of the most efficient and cost effective public health interventions and significant progress in reducing child mortality from VPDs has been made globally [4]. This study is the first of its kind for the population of DRC and provides an important initial step to better understand trends over time in childhood vaccination status. Using nationally representative data, we identified changes in overall vaccination coverage and its geographic variation over the past decade, both of which may help to inform future policy, interventions and research to improve vaccination rates among children in the DRC.

## Supporting information

S1 Table Weighted vaccination coverage estimates for the democratic republic of the Congo demographic and health survey respondents 12–23 months of age by survey year. (DOCX)
