# Supplementary material for: Changes in childhood vaccination coverage over time in the Democratic Republic of the Congo
Source: PLoS One. 2019 May 24;14(5):e0217426. doi: 10.1371/journal.pone.0217426 (PMC6534301; doi:10.1371/journal.pone.0217426)
Supplement: S1 Table — (DOCX) [file pone.0217426.s001.docx]

| **Supplemental Table 1.** Weighted vaccination coverage estimates for the Democratic Republic of the Congo Demographic and Health Survey respondents 12-23 months of age by survey year | | | | | |
| --- | --- | --- | --- | --- | --- |
|  | 2007  (n=2,238) | | 2013-2014 (n=4,772) | |  |
|  | n | % (95% CI) | n | % (95% CI) | *p-value* |
| Vaccination card^a^ |  |  |  |  |  |
| Yes, seen (1) | 337 | 15 (11-19) | 689 | 15 (12-17) | *0.005* |
| Yes, not seen (2) | 1,008 | 45 (41-50) | 1,861 | 39 (35-43) |  |
| No longer has card (3) | 193 | 9 (7-11) | 750 | 16 (14-8) |  |
| No card (0) | 687 | 31 (26-36) | 1,440 | 30 (27-34) |  |
| Reported vaccinations from vaccination card or maternal recall |  |  |  |  |  |
| BCG^b^ | 1,668 | 75 (70-80) | 4,011 | 84 (82-86) | *0.0003* |
| Polio0^c^ | 973 | 44 (38-49) | 2,172 | 46 (42-49) | *0.5596* |
| Polio1^d^ | 1,806 | 81 (77-85) | 4,251 | 92 (90-93) | *<0.0001* |
| Polio2^e^ | 1,596 | 72 (67-76) | 3,818 | 86 (84-88) | *<0.0001* |
| Polio3^e^ | 1,056 | 48 (43-52) | 2,921 | 66 (63-69) | *<0.0001* |
| Polio vaccination completion (OPV1-3)^e^ |  |  |  |  |  |
| Full (1) | 1,052 | 47 (43-52) | 2,919 | 66 (63-69) | *<0.0001* |
| Partial (2) | 751 | 34 (30-38) | 1,126 | 25 (23-28) |  |
| None (0) | 420 | 19 (15-23) | 378 | 9 (7-10) |  |
| DTP1^f^ | 1,616 | 74 (69-79) | 3,853 | 81 (79-84) | *0.0113* |
| DTP2^g^ | 1,371 | 63 (58-68) | 3,565 | 75 (72-78) | *<0.0001* |
| DTP3^g^ | 1,107 | 51 (46-56) | 2,980 | 63 (59-66) | *0.0003* |
| DTP vaccination completion (DTP1-3)^g^ |  |  |  |  |  |
| Full (1) | 1,103 | 51 (45-56) | 2,979 | 63 (59-66) | *0.0006* |
| Partial (2) | 513 | 24 (19-28) | 873 | 18 (16-21) |  |
| No (0) | 557 | 26 (21-31) | 889 | 19 (16-21) |  |
| Measles^h^ | 1,560 | 71 (66-76) | 3,756 | 79 (77-81) | *0.0013* |
| Yellow fever^i^ | 1,190 | 54 (49-59) | 3,443 | 73 (70-76) | *<0.0001* |
| Vaccination status^j^ |  |  |  |  |  |
| Full (1) | 548 | 26 (22-30) | 1,942 | 45 (41-48) | *<0.0001* |
| Partial (2) | 1,231 | 58 (53-62) | 2,130 | 49 (46-52) |  |
| None (0) | 357 | 17 (13-20) | 285 | 7 (5-8) |  |

^a^ 2007 n=2225; 2013 n=4741.

^b^ 2007 n=2214; 2013 n=4761.

^c^ 2007 n=2228; 2013 n=4763.

^d^ 2007 n=2226; 2013 n=4630.

^e^ 2007 n=2223; 2013 n=4423.

^f^ 2007 n=2174; 2013 n=4744.

^g^ 2007 n=2172; 2013 n=4741.

^h^ 2007 n=2198; 2013 n=4757.

^i^ 2007 n=2202; 2013 n=4739.

^j^ 2007 n=2136; 2013 n=4357.
